# Fluorescein-distribution in confocal laser endomicroscopy allows for discrimination between primary brain tumours and metastases

**DOI:** 10.3389/fsurg.2025.1567711

**Published:** 2025-04-28

**Authors:** Maria C. Brielmaier, Johannes Reifenrath, Franziska Ganster, Nicolas Pensel, Jens Gempt, Bernhard Meyer, Jürgen Schlegel, Arthur Wagner

**Affiliations:** ^1^ Department of Neurosurgery, Universitaetsklinikum Rechts der Isar, Technical University Munich, Munich, Germany; ^2^ Department of Neuropathology, Institute of Pathology, Technical University Munich, Munich, Germany; ^3^ Department of Neurosurgery, University Medical Center Hamburg-Eppendorf, University of Hamburg, Hamburg, Germany

**Keywords:** confocal laser endomicroscopy, sodium-fluorescein, brain-tumour, metastases, digital biopsy

## Abstract

**Introduction:**

Emerging digital biopsy technologies, such as confocal laser endomicroscopy (CLE), have shown how neuro-oncological surgery can be revolutionised with the help of rapid, intraoperative tissue assessment which offers a high diagnostic accuracy. For *in vivo* CLE of cerebral neoplasia, there is only one existing staining agent—Sodium-Fluorescein (SF)—approved for intravenous application. The staining characteristics of SF yet remain unclear.

**Methods:**

In order to understand the dyeing behaviour of SF, we initiated a pilot study, comparing the staining pattern when incubating established tumour cell lines with SF *in vitro* to the distribution of intravenously applied SF *in situ*—examined with CLE *ex vivo* as well *in vivo*.

**Results:**

*In vitro*, the cell lines showed a hyperbolic, time-dependent cellular accumulation of SF. Carcinoma cell lines showed significantly more intracellular SF than glioma cell lines. This phenomenon could be observed when applying SF intravenously before tumour resection surgery. In gliomas and meningiomas, SF could only be detected in 14.3% and 16.1% of images per case, while in carcinoma metastases, SF would accumulate in 68.1% of images per case.

**Discussion:**

The concordant results from *in vitro*, *ex vivo* and *in vivo* investigations could show that the cellular accumulation of the staining agent SF varies depending on the tumour entity. While primary brain tumours rarely show intracellular SF accumulation, carcinoma metastases display SF intracellularly more frequently. Therefore, SF allows for a better discrimination between brain tumours and brain metastases when performing CLE *in vivo*.

## Introduction

For several reasons, intraoperative diagnostics play a key role in the neurosurgical operating room. First and most importantly, the intraoperative diagnosis might influence the further surgical approach and the extent of resection. Secondly, the preoperative diagnoses for neurosurgical operations can be less accurate than in other surgical fields due to the lower number of preoperative biopsies, and MRI and CT scans are not always reliable in determining the different tumour entities.

The current standard method for intraoperative diagnostics is histopathology, performed by neuropathologists using frozen sections. The frozen section enables the neuropathologist to make a preliminary diagnosis, which can then be discussed with the neurosurgeon in the operation room. This procedure takes 20–40 min (1–3) and can serve as a basis for intraoperative decisions regarding the further operative approach.

In addition, the standard processing for diagnostics produces artefacts *per se* because it no longer deals with “living” tissue *in situ* due to the resection and fixation of the tissue. Thus, the demand for artifact-free imaging of tumour tissue *in vivo* can be met by different methods, for example with so-called “digital biopsies.” Digital biopsies allow the neurosurgeon to examine the living tissue *in situ* together with the neuropathologist, who can join remotely and discuss the images with the surgeon in real time.

As one of the most advanced digital biopsy techniques, confocal laser endomicroscopy (CLE) is based on confocal laser imaging technology, enabling the investigator to explore the tissue on a cellular level in real time and at different depth levels. The first and—so far—only fluorescent agent approved for intravenous application as a contrast agent for CLE of cerebral neoplasia is Sodium-Fluorescein (SF). The SF-guided CLE of brain tumours offers faster tissue assessment than conventional frozen sections (1, 3). Although the staining mechanism and kinetics of SF so far have only been studied in experimental brain tumour models, the distribution of SF enables neurosurgeons and neuropathologists to recognise cytoarchitectural features (4) and differentiate between different tumour histoarchitectures. Therefore, understanding the distribution of SF and its staining mechanisms might play a key role in the future impact of intraoperative diagnostics.

During our clinical *in vivo* CLE study (3), we observed a different cellular uptake of the staining agent in brain tumour cells vs. metastatic cells. The present investigation of the SF distribution *ex vivo* and *in vitro* aims to prove the diagnostic power of this phenomenon. Since discrimination between brain tumour entities and metastases is one of the most challenging decisions in conventional frozen section diagnostics (5), SF distribution might offer an opportunity to improve intraoperative differential diagnostics.

## Material and methods

### Distribution of sodium-fluorescein *in vitro*

To investigate the uptake of Sodium-Fluorescein (SF) in glioma cells and adenocarcinoma cells, respectively, glioma cell lines LN18, LN229, T98G, and U87MG and adenocarcinoma cell lines MCF7 (breast cancer), SW48 (colorectal cancer), MDA (breast cancer), and OV-MZ-6 (ovarian cystadenocarcinoma) were used. Cells were cultivated under standard cell culture conditions (37°C, 5% CO2) on round coverslips (Hartenstein, Germany, #DKR0).

To assess the time-dependent staining kinetics of SF in cell culture conditions, we incubated a monolayer of formaldehyde-fixed Ln229 cells with 1 mg/ml SF (1 mg/ml; Sigma-Aldrich #46960) after 0, 5, 10, and 20 min of incubation. F For staining of dead cells in the viability-dependent intensity analysis, specimens were first fixed with 4% formaldehyde supplemented with Hoechst at a concentration of 1:10,000 for 10 minutes before incubation with SF (1 mg/ml) and propidium iodide (1 μl/ml; Sigma-Aldrich #P4864). Inversely, viable cells were first incubated with 1 mg/ml SF for 30–40 min, and if applicable with propidium iodide, diluted in the cell culture medium prior to fixation. Subsequent epifluorescence imaging was performed on an Axio Imager microscope (Zeiss, Munich, Germany) with an HBO 100 lamp and imaged using the Axiocam monochrome camera (Axiocam 503 mono, Zeiss; 2.8 megapixel). Images were processed in ZEN lite (Zeiss, 3.8 software). Measurements for the time- and viability-dependent stains were repeated twice; all other experiments three times in independent samples.

Data analysis was performed using Matlab R2023b with the image processing toolbox for cell culture experiments. Briefly, we identified regions of interest (ROI) using the inbuilt imbinarize-method. We empirically determined a threshold value of 0.2 and a pixel count greater than 300. For each image, we measured the mean fluorescence intensity of each ROI. The final fluorescence intensity was as then computed as the median of all ROIs minus the background fluorescence intensity (all areas below a z-score of 0.2). In some pictures, we detected a non-cellular, extremely fluorescent debris that likely was undissolved SF. Pixels with an intensity count above 15,000 counts were thus excluded from further analysis. To compare different cell lines, the differential fluorescence intensity was calculated as the difference between the untreated control samples and the absolute fluorescence intensity of the samples incubated with SF for 30 min. Cell lines were compared using the Mann–Whitney-*U*-Test. *a priori*, a significance level of 0.05 was determined. Plots were drawn in SPSS (Version 29.0.2.0, IBM Corp, Armonk, USA).

### Distribution of sodium-fluorescein *in vivo*

In the following, we explored the distribution pattern of SF in living human CNS tissue by applying the fluorescent dye intravenously as previously described (3).

#### Confocal laser endomicroscopy *ex vivo* and conventional fluorescence microscopy

In a first small case series, we examined the SF-stained brain tumour tissue after resection to gain insight into the SF staining pattern *in vivo*. Fluorescein Alcon® (10% solution for injection, 5 ml, Alcon AG) was used as staining agent. Its water-soluble sodium-salt Sodium-Fluorescein (SF) was applied intravenously at a dosage of 5 mg per KG bodyweight during the dura incision. When resecting tumour tissue for conventional frozen sections, additional tissue was resected to examine the SF distribution. This tissue was immediately examined *ex vivo* with the CONVIVO® microscope (Carl Zeiss Meditec AG, Oberkochen, Germany), which offers a laser power of 1 mW and a wavelength of 488 nm. Its field of view measures 475 μm × 267 μm with an image resolution of 1,920 × 1,080 pixels (full HD) and a frame rate up to 2.35 frames per second. Further specimens were resected from the cerebral lesion for an investigation with a conventional fluorescence microscope.

These specimens were snap frozen, sliced on a cryostat, stained with conventional Hematoxylin & Eosin (H&E) and on the corresponding following slide, conventional fluorescence microscopy was performed to detect the SF fluorescence with a multichannel fluorescence microscopy (Imager Z2, Carl Zeiss Meditec AG). The comparison of SF- with H&E-stained slices allowed the determination of the borders of the cell bodies and, therefore, the differentiation between intracellular and extracellular SF accumulation.

#### Confocal laser endomicroscopy *in vivo*

Finally, we investigated the distribution of intravenously applied SF *in situ* with CLE. The materials and methods have previously been described (3). Briefly, Fluorescein Alcon® was applied at the start of the surgery, approximately 20–40 min before imaging with CLE, and all cases were investigated with a CONVIVO® microscope. CLE was performed *in vivo* by the neurosurgeon, who applied the scanning probe to the surface of the lesion after cutting open the dura. Single images and stack images were taken by the surgeon with the assistance of a second person operating the microscope body and adjusting the imaging parameters and a neuropathologist analyzing the images via a remote workplace connection (3).Over the course of the clinical trial the imaging parameters were chosen manually in each respective case, but consistent with previously set up ranges. The imaging depth was set between 40 μm and 150 μm and and for laser power and contrast adjustment we used the automated setting, where the device sets the imaging parameters itself.

For the present study, all *in vivo* CLE-acquired images were manually re-analysed by two individual evaluators. Therefore, a binary assessment was conducted to determine the presence or absence of cellular SF enrichment in each image. Images displaying round, bright structures against a dark background were classified as positive for cellular SF enrichment. In contrast, images with a bright background and darker structures in the foreground were classified as negative. For each case, both the absolute and relative proportions of images per case showing cellular SF accumulation were calculated and compared between brain tumour entities (glioma and meningioma) and metastases.

## Results

### Distribution of sodium-fluorescein *in vitro*

#### Time- and viability-dependent staining characteristics of Sf in Ln229 cells

**Figure 1:**
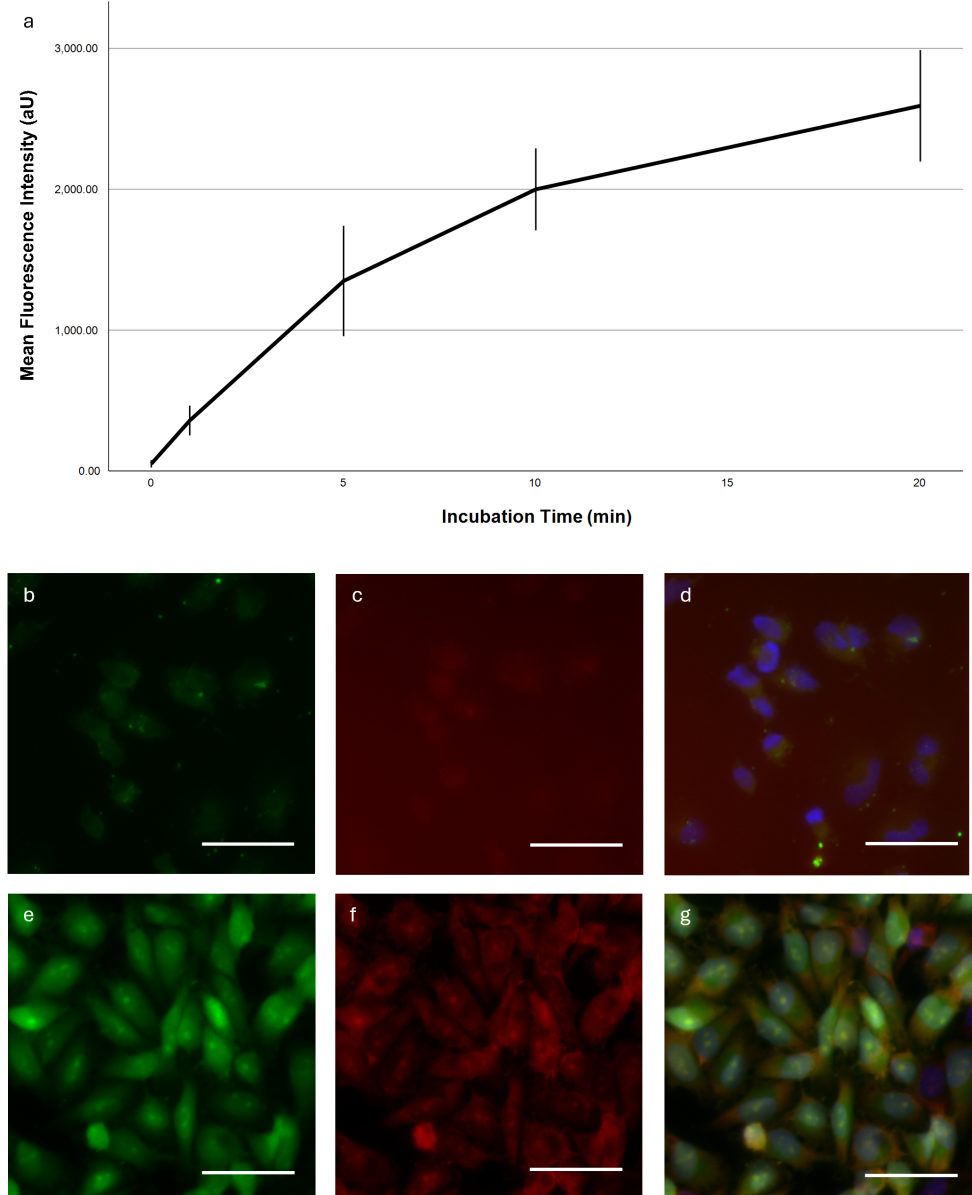
Time-dependent fluorescence intensity and representative microscopy images. **(a)** Mean fluorescence intensity in Ln229 cells as a function of incubation time. Error bars indicate the 95% confidence interval. **(b–g)** Fluorescent images of a Ln229 cell monolayer stained with SF **(b,d)**, PI **(c,f)** and Hoechst **(d,g)** for 40 min. Exposure time was adjusted for each picture individually. Dynamic display range was set to the minimum/maximum non-zero value for the green and DAPI channels; for the red channel the maximum pixel value was set to the camera's maximum pixel value of 16,384. White bar ∼50 μm. **(a–d)** Incubation of living cells, **(e,f)** Incubation of fixed cells.

For the time-dependent staining characteristic we measured a hyperbolic intensity increase from 50.80 units (95%-CI: 24.53–77.07) in the minimally autofluorescent control group up to 2,592.70 units (2,197.36–2,988.04) at 20 min (Figure 1a). After that, we detected no further intensity increase in dead or vital cells (data not shown).

We then compared the SF accumulation in viable and dead Ln229 cells (Figures 1b–g). The latter showed much greater fluorescence intensities overall and distinct staining of the entire cell with a prominent nucleolar intensity peak. Viable cells were less intensely fluorescent and did not show a nucleolar staining. Importantly, the SF distribution in dead cells correlated with the propidium iodide signal.

#### Comparison of the fluorescence intensity between different cell lines

**Figure 2:**
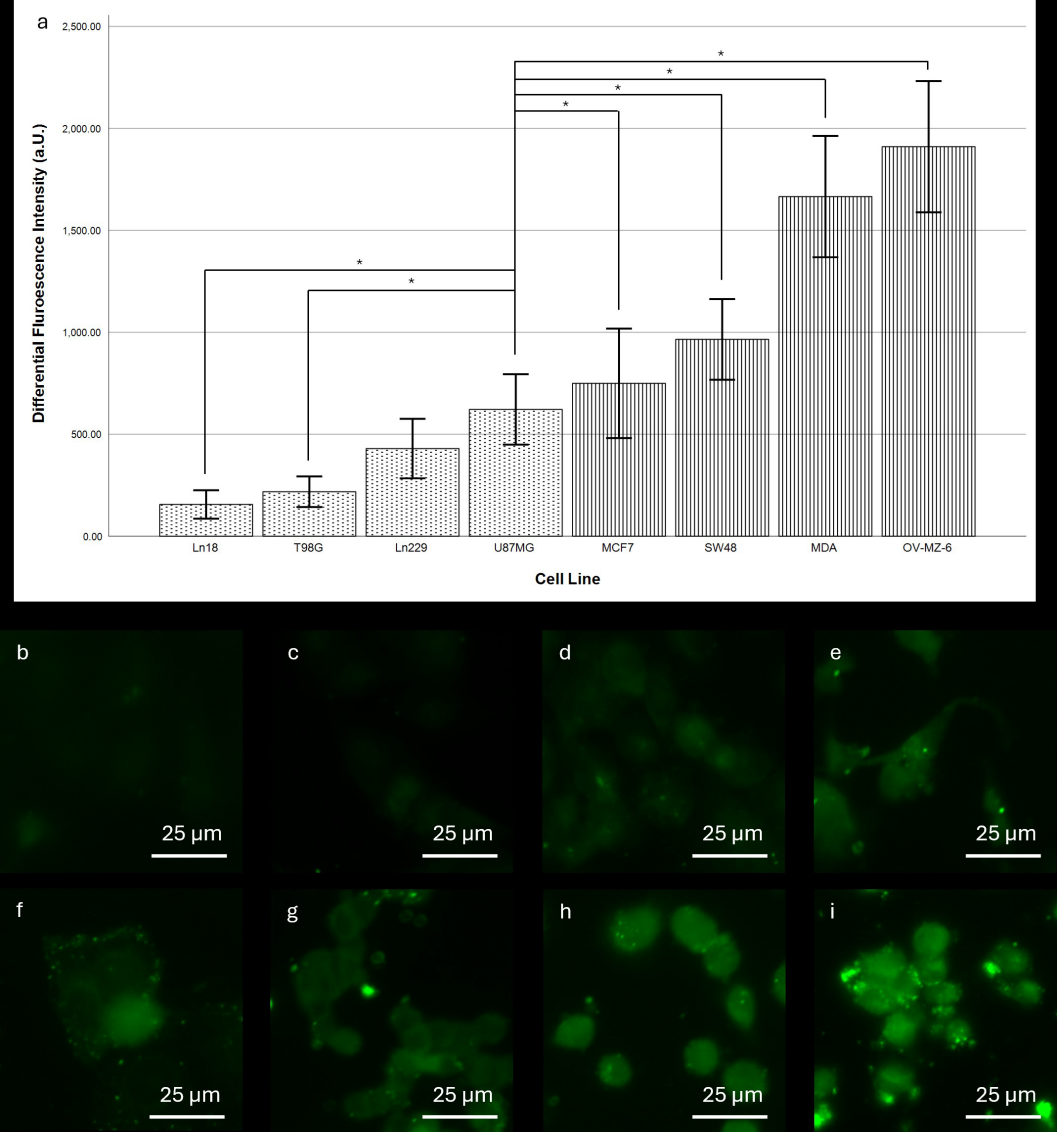
Comparison of fluorescence intensity between different cell lines after SF stain. **(a)** Differential fluorescence intensity per cell line. Dotted pattern: glioma cell lines, striped pattern: adenocarcinoma cell lines. Error bars indicate the 95% confidence interval. *indicates a significance level of 0.05. For cell lines Ln18, T98G, and Ln229 differences to all of the adenocarcinoma were significant at the 0.05 level. **(b–i)** Representative fluorescence microscopy images of the stained cell lines: **(b)** Ln18, **(c)** T98G, **(d)** Ln229, **(e)** U87MG, **(f)** MCF7, **(g)** SW48, **(h)** MDA, and **(i)** OV-MZ-6. Scale bars: 25 µm.

The subsequent cell culture experiments confirmed distinctly different fluorescence intensities for different tumour entities. Notably, all glioma cell lines except U87MG exhibit significantly lower fluorescence intensities than the adenocarcinoma cell lines (Figure 2). Among the glioma cell lines, Ln18 shows the weakest intensity with 155.99 units (95%-CI: 86.28–255.70), while U87MG cells show a mean differential fluorescence intensity at 621.88 units (95%-CI: 449.49–794.64). For adenocarcinoma cell lines the lowest fluorescence intensity was observed in MCF7 cells with a mean of 749.83 (95%-CI: 481.49–1,018.17); the maximum intensity difference in OV-MZ-6 cells (1,910.23, 95%-CI: 1,588.29–2,232.17). The intensity difference between MCF7 and U87MG cells was not significant. The differences between all other adeno- and glioma cell lines were significant.

**Figure 3:**
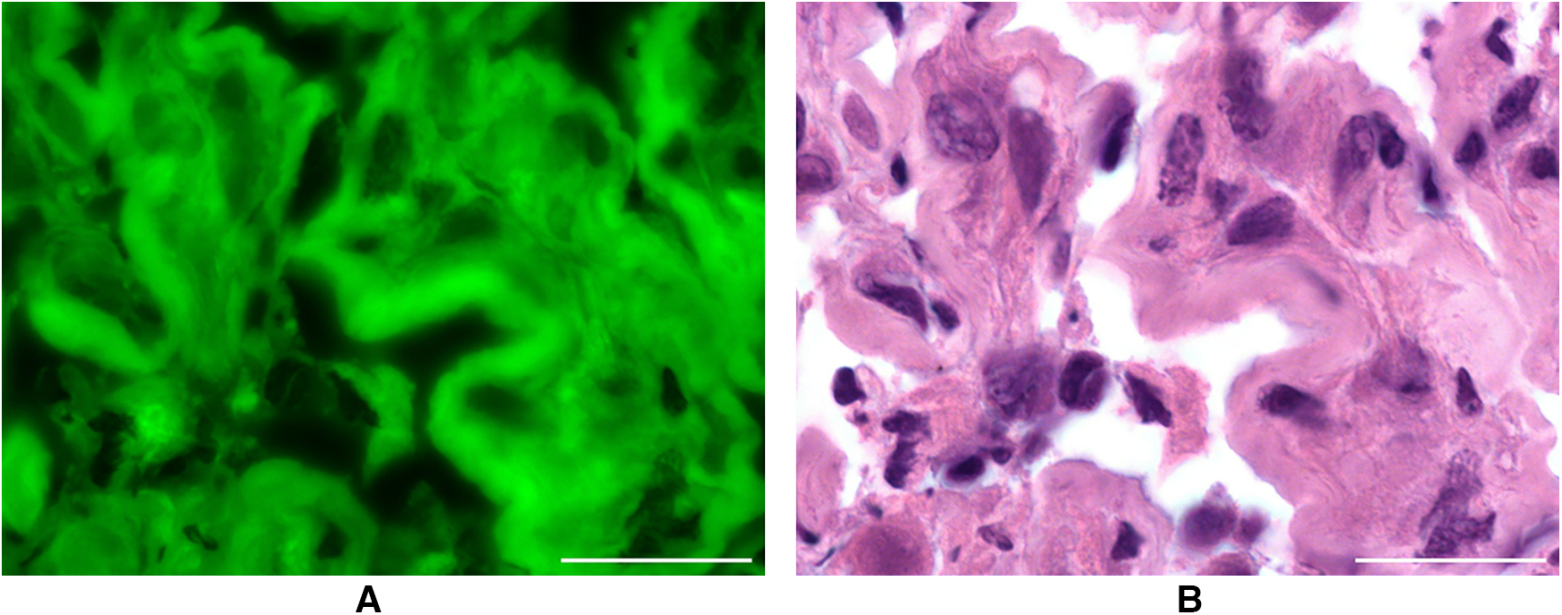
**(A)** resected metastasis after intravenous application of SF in conventional fluorescence microscopy. Scale bar: 50 µm. **(B)** H&E staining of the same metastasis as a comparison. Scale bar: 50 µm.

### Distribution of sodium-fluorescein *in vivo*

#### Confocal laser endomicroscopy *ex vivo* compared to conventional fluorescence microscopy

In this small case series, 13 cases were investigated with confocal laser endomicroscopy *ex vivo*. In addition to the readability of the images and the accumulation of dye in the different tumour zones, the structures stained by SF were investigated. To achieve this, SF-guided CLE images of the tumour were compared with H&E-stained sections of the same sample, which were examined under the fluorescence microscope (see Figures 3A,B).

In 11 out of 13 patients (84.62%), a match was found between the tumour structures on CLE images and in conventional fluorescence microscopy. In two samples, it was not possible to compare the images.

The tissue could be visualised under the fluorescence microscope in all 13 cases without additional fluorescence staining of the H&E slides. The structures suspected as tumour cells in the CLE images with a dark centre and light border were identified as such by fluorescence microscopy. Sodium fluorescein stained the cytoplasm of the pathologically altered cells, but most of their nuclei did not accumulate any SF.

Differences were shown regarding the dye affinity of common tumour entities for SF. In this case series, metastases and meningiomas took up SF particularly well. Sharp images could be produced that showed the cytomorphology well. Examination of the CLE images of glioblastomas WHO CNS° 4 revealed a diffuse appearance. There are areas of different staining intensity. The investigations have also shown that oligodendrogliomas WHO CNS° 3 absorb the dye less strongly than the other tumour entities.

#### Confocal laser endomicroscopy *in vivo*

When investigating the fluorescent dye SF *in vivo* with CLE, the full analysis set (FAS) consisted of 111 cases with a total sum of 4,508 images. The patient collective included an almost even amount of male (*n* = 53, 48%) and female patients (*n* = 58, 52%). The patients were aged from 18 to 87, with an average of 59.9 ± 16.9 years. The final histology of the FAS showed the following entities: gliomas (*n* = 52, 47.8%), meningiomas (*n* = 22, 19.8%), carcinoma metastases (*n* = 23, 20.7%) and other diagnoses (*n* = 14, 12.6%). Other diagnoses were: cavernoma (*n* = 2), gliosarcoma (*n* = 2), necrosis (*n* = 2), reactive inflammation (*n* = 2), subependymoma (*n* = 1), craniopharyngioma (*n* = 1), colloid cyst (*n* = 1), myxoid glioneuronal tumour (*n* = 1), teratoma (*n* = 1) and infarction (*n* = 1).

In its entirety, the FAS consisted of 4,508 images, with an average number of 40.6 *in vivo* CLE images taken per case. 40.9% (*n* = 1,846) of all images of the FAS were useable for diagnostic purposes. 59.1% (*n* = 2,662) were not useable due to insufficient image quality. The most common impairments of image quality were motion artefacts, haemorrhage, blurriness and low contrast levels.

**Figure 4:**
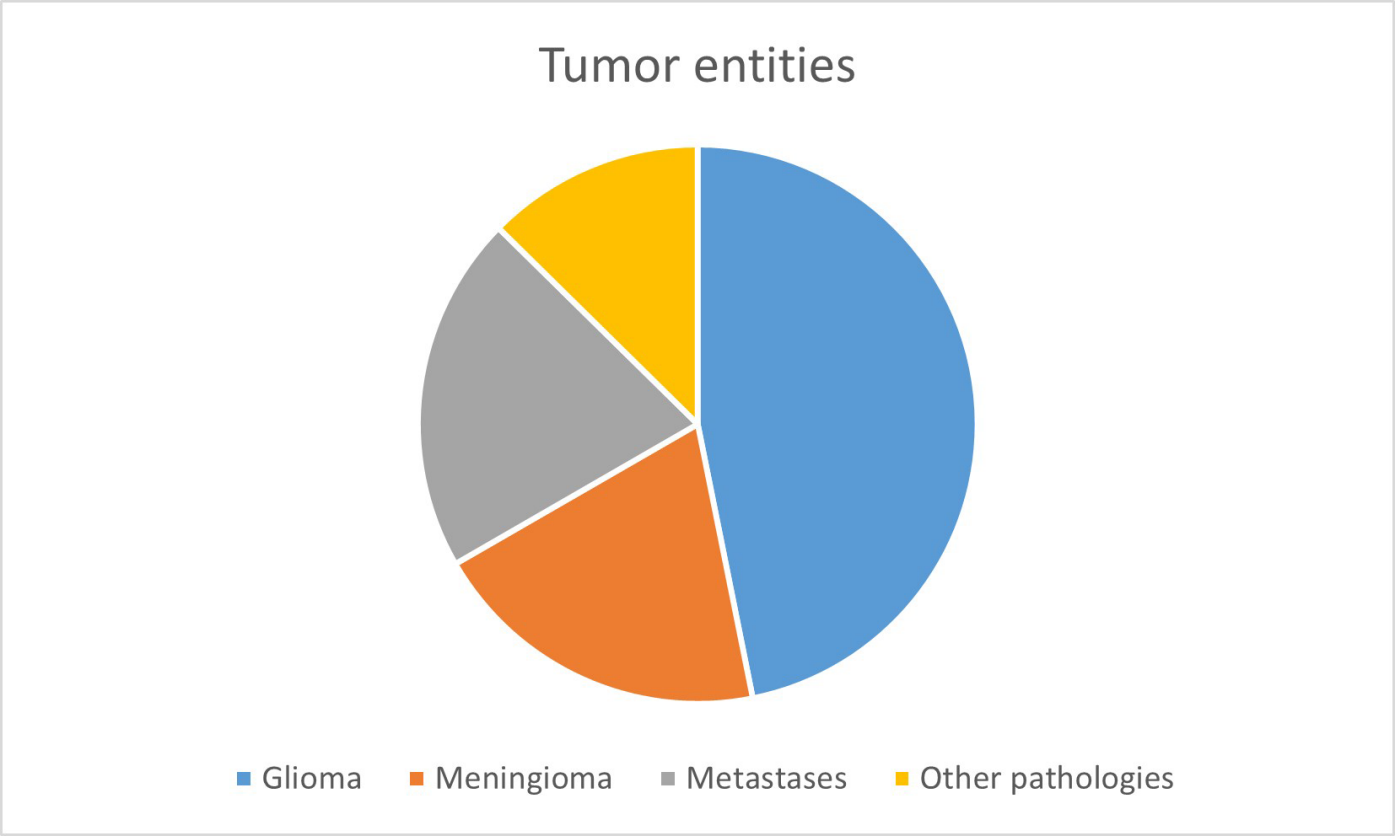
The different entities which were investigated with SF-guided CLE *in vivo*.

**Figure 5:**
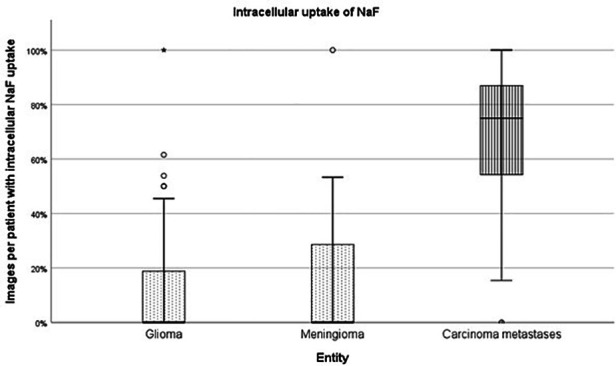
The images of each case were analysed for SF distribution, the proportion of images per patient with intracellular NaF uptake was calculated and compared between the different tumour entities.

**Figure 6:**
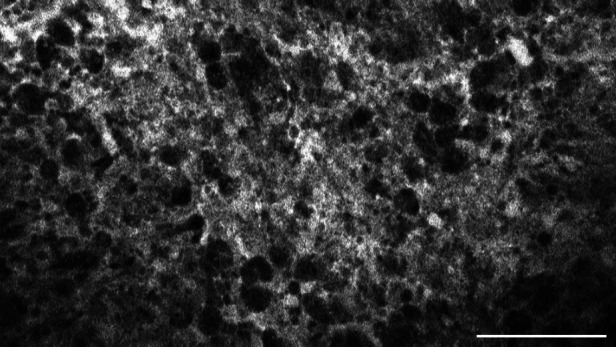
Glioblastoma multiforme, WHO CNS°4, IDH wild type, examined *in vivo* with CLE, shows diffuse accumulation of SF in the extracellular matrix. Scale bar: 100 µm.

**Figure 7:**
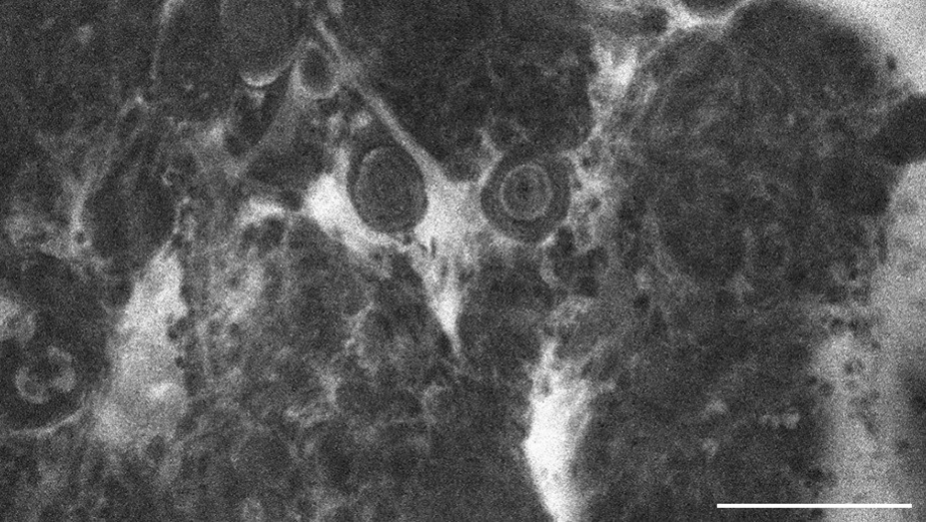
Transitional meningioma, WHO CNS°1, examined *in vivo* with CLE, shows diffuse extracellular SF-accumulation as well. Scale bar: 100 µm.

**Figure 8:**
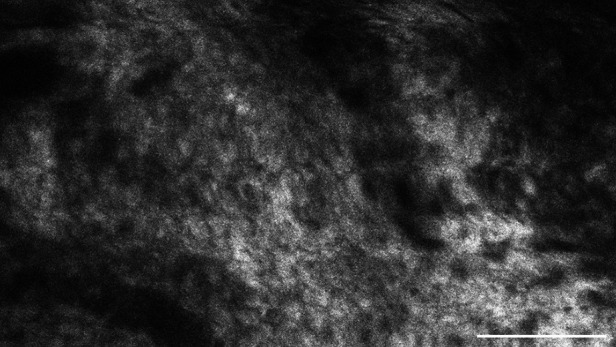
This metastasis of a squamous cell carcinoma, examined *in vivo* with CLE, shows intracellular accumulation of the contrast agent sodium-fluorescein. Scale bar: 100 µm.

The FAS, consisting of 111 cases with a total of 4,508 images conducted with CLE *in vivo,* was analysed for the cellular uptake of SF. Due to the high heterogeneity of the fourth diagnostic group, “other diagnoses,” only the entities gliomas, meningiomas and carcinoma metastases were compared to each other (see Figure 4). The 2,662 diagnostically not useable images were taken out of consideration. Per each case, the percentage of images that showed cellular SF accumulation was calculated and compared between the different tumour entities, as shown in Figure 5. Results showed that gliomas and meningiomas rarely demonstrated SF intracellularly (see Figures 6, 7). On average, 2.7 images (14.3% ± 23.9%) per case in glioma and 2.7 images (16.1% ± 24.9%) per case in meningioma indicated cellular SF accumulation. In comparison to that, carcinoma metastases showed much higher cellular uptake of SF with an average of 9.1 images (68.1% ± 27.1%) per case (see Figure 8).

## Discussion

### Interpretation of the results

Many modern digital methods for intraoperative microscopic diagnostics are based on fluorescence uptake in the tumour tissue. Confocal laser endomicroscopy (CLE) is a relatively new approach in neurosurgery and utilizes the uptake of sodium-fluorescein (SF). The cellular distribution of SF is one of the most important diagnostic features, but so far, it has only sparely been investigated. Therefore, this pilot study aimed to investigate the distribution pattern of the staining agent SF to gain a better understanding of the images acquired intraoperatively with CLE *in vivo*.

### Interpretation of the findings in the *in vitro* experiments

In the first experimental approach, we investigated the uptake into glial tumour cells and compared it to the uptake into tumour cells derived from adenocarcinomas. We observed clear differences between the different tumour entities. In accordance with previous literature (6), glioma cells exhibited only weak fluorescence signals, while adenocarcinoma cell lines demonstrated significantly stronger intensities. This also fits with the kinetics of SF uptake since adenocarcinoma cells showed a significantly quicker uptake. Interestingly, U87MG cells were the brightest glioma cell lines tested and the only ones with stem cell properties. Though the exact mechanisms of SF uptake remain unknown, evidence suggests that transmembrane channels might play a key role since monocarboxylate transporters have been shown to pass SF into corneal epithelial cells (7).

In our *in vitro* experiments, we elucidate major staining characteristics of SF. We identify the staining kinetics as logarithmic with a levelling out of the curve after 20 min. This corroborates previous research describing similar kinetics for the accumulation of SF in rodent brains, peaking at 30 minutes post-injection (8). However, logarithmic staining kinetics are not unique to SF and have also been observed for indocyanine green (9) and nanoparticles (10).

We further demonstrate that SF accumulates preferably in dead cells but is mainly excluded from living glioma cells. Using propidium iodide, a fluorescent tracer that can only cross damaged cell membranes, we relate the intracellular accumulation of SF to an impairment of the membranous integrity. The impermeability of membranes to fluorescein has been observed previously in epithelial cells (11) and is an underlying principle of the fluorescein diacetate assay (12). However, recent studies have also shown intracellular SF in leukocytes and tumour cells (13).

In the cell culture experiments we did not investigate meningioma cell lines because meningioma can usually be easier determined preoperatively in MRI e.g., than glioma or metastases. Frequently, the differentiation between glioma and metastases is quite difficult with preoperative imaging. Furthermore, glioma and carcinoma cell lines are easier to cultivate and experiment with, another reason why we decided to not use meningioma cell lines for the SF distribution analysis *in vitro*.

### Interpretation of the findings from CLE *ex vivo* and *in vivo*

These results are consistent with our findings from the distribution of intravenously applied SF in intracranial lesions analysed with CLE. For reference, we used conventional fluorescence microscopy *ex vivo*. By comparing images generated with CLE *ex vivo* with conventional fluorescence microscopy, we could prove that SF can accumulate intracellularly in the tumour tissue after intravenous injection of the fluorescent dye. Since we only investigated 13 cases with this elaborate method, we could not clearly distinguish between the different staining behaviours of the various entities.

Next, we collected 111 cases from our recent CLE *in vivo* clinical trial to investigate the distribution of SF *in vivo*, and we observed consistent results in these cases that aligned with our initial *in vitro* experiments.

The tumour entities derived from brain tissue, glioma and meningioma, rarely showed cellular accumulation of SF. On average, in only 14.3% (glioma) to 16.1% (meningioma) of the images per case SF could be visualized within the cell body. Instead, the staining agent was mainly distributed in the extracellular matrix and in the blood. This created a brightly glowing background from which the cell bodies stood out darkly.

In comparison to that, metastases frequently showed SF in the cell body. On average, 68.1% of the images per case suggested intracellular SF accumulation. This feature distinguishes the metastases from brain tumours, although it is not yet clear why the tumour entities have this different cellular uptake of SF. Other studies have acknowledged the different intracellular SF uptake in different tissue types as well (14). They indicate that glioma rarely show SF in the cytoplasm *in vivo*, but that some *ex vivo* glioma specimen could acquire intracellular fluorescence due to cell membrane decay in dead cells. Furthermore, intracellular SF uptake does not seem to be a phenomenon restricted only to carcinoma cells. This phenomenon can also occur *in vivo* in other cell types, as intracellular SF uptake has been documented in pituitary adenoma cells as well, for example (15).

In this pilot study, for the first time ever, SF-distribution was examined in combination with *in vivo*, *ex vivo*, and *in vitro* methods*.* By comparing the results, we could prove that glioma and meningioma, brain-derived tumour entities, and metastases show a different distribution of the staining agent. While glioma and meningioma show less intracellular uptake of SF, the metastases frequently presented with cellular accumulation of SF.

### Study limitations

Firstly, the experiments *in vitro* come with a few limitations. An impairment of this pilot study might be that, although CLE *in vivo* was performed in numerous cases with a high variety of tumour entities—especially glioma, meningioma and metastases, the experiment *in vitro* was obtained with only seven cell lines, glioma as well as metastases. Meningioma cell lines were not included as meningioma cells rarely grow under standard cell culture conditions.

In addition, we show that SF and Eosin are similar in their staining affinity to histoarchitectural features. This can likely be explained by the structural similarity since Eosin is a derivative of SF. Nevertheless, we observed slight differences in the image contrast and dynamic display range of the eosin and SF images. We attribute this to the technical limitations of our experimental setup, mainly the different illumination modes for Eosin and SF.

Secondly, the examination of CLE *in vivo* also shows some limitations. The dichotomous differentiation between images with and without cellular accumulation of SF could not be validated *in vivo* as well, since there is currently no other existing staining agent approved for intravenous application and *in situ* CLE imaging. In the future, there will be a need for further staining agents, which can be applied intravenously or topically, to perform CLE *in vivo*.

Furthermore, the analysis of the CLE images *in vivo* was obtained by the examiners who had to interpret all images by hand since there is currently no existing automatic image interpretation algorithm which can determine cellular vs. extracellular SF accumulation in CLE images. Although the examiners analysed all images in a single-blinded manner, without access to any diagnostic or background information of each respective case, there is still a risk of personal bias. Especially, since CLE for the *in vivo* use is a very new technology, it comes with a long learning curve. Therefore, further investigations to determine the interrater-bias would certainly be important.

In addition, the image quality when performing CLE *in vivo* can be impaired by imaging artefacts such as movement artefacts or haemorrhage. Movement artefacts can impair the examiner's ability to interpret the silhouette of the cell body and haemorrhage, containing high amounts of SF in the plasma, could be mistaken for SF accumulation in the extracellular matrix of the tumour tissue. Beyond that, bleeding artefacts can be misinterpreted, as erythrocytes appear as black cells against an intensely SF-stained background. Hence, images with insufficient image quality were ruled out before performing the analysis of SF distribution. In the future, we would like to investigate the use of AI-driven algorithms to rule out CLE images with insufficient image quality to offer even faster, more accurate intraoperative assessment of tissue. On further notice, since we worked with individually set image parameters in each case, it cannot be ensured that all images are entirely comparable to one another in terms of image quality and SF enhancement.

In the near future, an even more objective analysis could be performed by using algorithms ruling out any subjective mistakes. There are already existing algorithms that enhance the histopathological interpretation of CLE images in gastric cancer (16). In neuro-oncological research, great progress has recently been made in experiments with AI-driven algorithms as well. For example, DCNN (deep convolutional neural networks) can be used to determine whether a CLE-aquired image has sufficient image quality or to differentiate between tumour, injury and normal tissue (17, 18). Moreover, weakly supervised learning algorithms are able to identify regions of interest or typical histological features in CLE images with a high accuracy (19). Therefore, in the near future, computer algorithms enable the interpretation of larger image volumes with less manual work and less inter-rater bias.

Although this investigation might show minor impairments, the results hint to a fruitful impact for the future of CLE of cerebral neoplasia. According to our findings, intracellular SF-accumulation is more likely in metastatic lesions than in brain tumours. The results above prove *in vivo* CLE to be a great addition to the neurosurgical operating room, as SF-guided *in vivo* CLE offers the possibility to better differentiate between certain tumour entities. Especially neuroradiologically contrast-enhanced lesions are difficult to determine. Differential diagnoses of tumours which are hyperintense in contrast-enhanced MRI imaging include highly malignant glioma as well as carcinoma metastases. In this situation, the SF-distribution allows for a better discrimination between the two diagnoses. Thus, SF-guided CLE can play an important role for fast intraoperative decision-making in neurosurgery.

The concordant distribution patterns *in vivo*, *ex vivo* and *in vitro* hint to different cell wall channels that differ in their ability to take up SF into the intracellular matrix. This difference is not only important for quick assessment of CLE images. It also opens the possibility for further studies on ion channels in neurooncology that could play a role in future patient-centred therapy options.

## Conclusion

The objective of this pilot study was to analyse SF-distribution patterns for a better understanding of images generated with CLE *in vivo*. The concordant results from the analysis of tumour cell lines *in vitro*, resected tumour tissue *ex vivo* and CLE-investigated tumour tissue *in vivo* all show a different intracellular SF uptake in brain-derived tumours compared to metastases. These findings are consistent with the existing literature on SF-distribution investigated *in vitro* as well as *in vivo*. Therefore, SF-guided CLE promises to improve intraoperative decision-making, especially when it comes to differentiating between high-grade glioma and carcinoma metastases, one of the most difficult intraoperative decisions so far. In addition to that, several other publications have proven the worth of CLE for intraoperative digital biopsies. They made its fast imaging process and its high diagnostic correctness accountable. SF-guided differentiation between malignant glioma and brain metastases only plays a minor role in the complexity of CLE image interpretation. Other important factors remain the image quality and the examined tissue region. By trying to diminish error sources, such as movement, haemorrhage and mishandling of the laser parameters, an even better image quality and better diagnostic accuracy might be achieved in the future.

However*, in vivo* CLE is not only a promising technology for identifying tumour entities via digital biopsies; it could also be used in different user scenarios. Since acquiring a tumour resection as far as possible without harming healthy brain tissue increases survival and quality of life for patients with glioblastoma multiforme CLE might be a game-changing technology for identifying the tumour margin and achieving a full resection of the lesion (20).

Furthermore, together with other contrast agents than SF, CLE might be able to achieve sharper images with less diffuse contrast-enhancements. Other contrast agents, e.g., indocyanine green and aminolevulinic acid (5-ALA), have long been used for macroscopical identification of lesions but could serve as contrast agents for *in vivo* use of CLE as well.

The short imaging time, the high diagnostic accuracy, and the improved decision-making in complex differential diagnoses prove the further worth of experimenting with CLE technology in the neurosurgical operating room.

## Data Availability

The raw data supporting the conclusions of this article will be made available by the authors, without undue reservation.

## References

[1] Abramov I Park MT Gooldy TC Xu Y Lawton MT Little AS Real-time intraoperative surgical telepathology using confocal laser endomicroscopy. Neurosurg Focus. (2022) 52(6):E9. 10.3171/2022.3.Focus2250 35921184

[2] Fotteler ML Liesche-Starnecker F Brielmaier MC Schobel J Gempt J Schlegel J Socio-organizational impact of confocal Laser endomicroscopy in neurosurgery and neuropathology: results from a process analysis and expert survey. Diagnostics (Basel. (2021) 11:11. 10.3390/diagnostics11112128 34829475 PMC8623423

[3] Wagner A Brielmaier MC Kampf C Baumgart L Aftahy AK Meyer HS Fluorescein-stained confocal laser endomicroscopy versus conventional frozen section for intraoperative histopathological assessment of intracranial tumors. Neuro Oncol. (2024) 26(5):922–32. 10.1093/neuonc/noae006 38243410 PMC11066924

[4] Belykh E Miller EJ Carotenuto A Patel AA Cavallo C Martirosyan NL Progress in confocal laser endomicroscopy for neurosurgery and technical nuances for brain tumor imaging with fluorescein. Front Oncol. (2019) 9:554. 10.3389/fonc.2019.00554 31334106 PMC6616132

[5] Kamp MA Santacroce A Zella S Reichelt DC Felsberg J Steiger HJ Is it a glioblastoma? In dubio pro 5-ALA!. Acta Neurochir (Wien). (2012) 154(7):1269–73. 10.1007/s00701-012-1369-2 22576268

[6] Diaz RJ Dios RR Hattab EM Burrell K Rakopoulos P Sabha N Study of the biodistribution of fluorescein in glioma-infiltrated mouse brain and histopathological correlation of intraoperative findings in high-grade gliomas resected under fluorescein fluorescence guidance. J Neurosurgery. (2015) 122(6):1360–9. 10.3171/2015.2.JNS132507 25839919

[7] Sun Y-C Liou H-M Yeh P-T Chen W-L Hu F-R. Monocarboxylate transporters mediate fluorescein uptake in corneal epithelial cells. Invest Ophthalmol Visual Sci. (2017) 58(9):3716–22. 10.1167/iovs.16-20998 28738415

[8] Folaron M Strawbridge R Samkoe KS Filan C Roberts DW Davis SC. Elucidating the kinetics of sodium fluorescein for fluorescence-guided surgery of glioma. J Neurosurg. (2019) 131(3):724–34. 10.3171/2018.4.jns172644 30192200 PMC6995036

[9] von Kroge P Russ D Wagner J Grotelüschen R Reeh M Izbicki JR Quantification of gastric tube perfusion following esophagectomy using fluorescence imaging with indocyanine green. Langenbecks Arch Surg. (2022) 407(7):2693–701. 10.1007/s00423-022-02546-0 35581393 PMC9640410

[10] Åberg C. Kinetics of nanoparticle uptake into and distribution in human cells. Nanoscale Advances. (2021) 3(8):2196–212. 10.1039/d0na00716a 36133761 PMC9416924

[11] Romanchuk KG. Fluorescein. Physicochemical factors affecting its fluorescence. Surv Ophthalmol. (1982) 26(5):269–83. 10.1016/0039-6257(82)90163-1 7046118

[12] Leenen EJTM Boogert AA van Lammeren AAM Tramper J Wijffels RH. Quantitative characterization of viability and growth dynamics of immobilized nitrifying cells. In: Wijffels RH Buitelaar RM Bucke C Tramper J, editors. Progress in Biotechnology, volume 11. Noordwijkerhout: Elsevier (1996). p. 341–8. 10.1016/S0921-0423(96)80046-3

[13] Musca B Bonaudo C Tushe A Battaggia G Russo MG Silic-Benussi M Sodium fluorescein uptake by the tumor microenvironment in human gliomas and brain metastases. J Neurosurg. (2024) 140(4):958–67. 10.3171/2023.7.JNS23873 37657099

[14] Belykh E Zhao X Ngo B Farhadi DS Byvaltsev VA Eschbacher JM Intraoperative confocal Laser endomicroscopy ex vivo examination of tissue microstructure during fluorescence-guided brain tumor surgery. Front Oncol. (2020) 10:599250. 10.3389/fonc.2020.599250 33344251 PMC7746822

[15] Belykh E Ngo B Farhadi DS Zhao X Mooney MA White WL Confocal Laser endomicroscopy assessment of pituitary tumor microstructure: a feasibility study. J Clin Med. (2020) 9(10):3146. 10.3390/jcm9103146 33003336 PMC7600847

[16] Cho H Moon D Heo SM Chu J Bae H Choi S Artificial intelligence-based real-time histopathology of gastric cancer using confocal laser endomicroscopy. NPJ Precis Oncol. (2024) 8(1):131. 10.1038/s41698-024-00621-x 38877301 PMC11178780

[17] Izadyyazdanabadi M Belykh E Mooney MA Eschbacher JM Nakaji P Yang Y Prospects for theranostics in neurosurgical imaging: empowering confocal Laser endomicroscopy diagnostics via deep learning. Front Oncol. (2018aa) 8:240. 10.3389/fonc.2018.00240 30035099 PMC6043805

[18] Izadyyazdanabadi M Belykh E Mooney MA Martirosyan N Eschbacher J Nakaji P Convolutional neural networks: ensemble modeling, fine-tuning and unsupervised semantic localization for neurosurgical CLE images. J Vis Commun Image Represent. (2018) 54:10–20. 10.1016/j.jvcir.2018.04.004

[19] Izadyyazdanabadi M Belykh E Cavallo C Nakaji P Preul MC Yang Y. Weakly-Supervised learning-based feature localization for confocal Laser endomicroscopy glioma images. 21st International Conference—part II Medical Image Computing and Computer Assisted Intervention—MICCAI; Granada, Spain (2018). (accessed September 16–20, 2028)

[20] Abramov I Mathis AM Xu Y On TJ Belykh E Mignucci-Jimenez G Intraoperative confocal laser endomicroscopy during 5-aminolevulinic acid-guided glioma surgery: significant considerations for resection at the tumor margin. J Neurosurg. (2024) 142(2):1–14. 10.3171/2024.5.Jns24140 39332037

